# Optimised murine model of influenza-induced respiratory sepsis for studying acute kidney injury

**DOI:** 10.1186/s42826-026-00294-6

**Published:** 2026-08-26

**Authors:** Yaqing Jiao, Will Lung Chan, Yuee Cai, Arthur Chuxi Liu, Yilin Zhang, John M. Nicholls, Timothy H. Rainer

**Affiliations:** 1 Department of Emergency Medicine, School of Clinical Medicine, Li Ka Shing Faculty of Medicine, The University of Hong Kong, Hong Kong SAR, China; 2 Department of Pathology, School of Clinical Medicine, Li Ka Shing Faculty of Medicine, The University of Hong Kong, Hong Kong SAR, China

**Keywords:** AKI, Influenza, Murine model, NGAL, Respiratory sepsis

## Abstract

**Background:**

Viral respiratory sepsis, driven by influenza and COVID-19, is increasingly prominent clinically. However, there is a lack of preclinical models that reliably replicate its associated acute kidney injury (AKI), a major contributor to morbidity and mortality. To address this gap, we refined an established model of influenza-induced respiratory sepsis by administering graded intranasal H1N1 A/PR/8/34 doses (3.7 × 10¹, 3.7 × 10³, and 3.7 × 10⁴ TCID₅₀) in male BALB/c mice. We defined humane endpoints at ≥ 30% body weight loss, monitored clinical severity, weight, and glycaemia daily for 14 days, and assessed multi-organ dysfunction via serum biochemistry, histopathology, renal qPCR, and longitudinal serum neutrophil gelatinase-associated lipocalin (NGAL) ELISA.

**Results:**

The 3.7 × 10⁴ TCID₅₀ dose yielded 66.7% mortality by day 8, with a peak clinical score (MSS) of 10, > 30% weight loss, and hypoglycaemia (blood glucose < 70 mg/dL). Infected mice exhibited dose-dependent multi-organ dysfunction, with substantial elevations in serum creatinine (median: 204 [IQR: 142–600] µmol/L), bilirubin (40.62 [IQR: 29.3–124.9] µmol/L), and creatine phosphokinase (CPK) (9822 [IQR: 1,272–11,352] U/L). Renal NGAL expression increased up to 7-fold, aligning with rising serum creatinine levels and histopathological evidence of glomerular enlargement and tubular degeneration. Serum NGAL showed early elevation (up to 4,990 ng/mL on day 2) in severely affected mice, with levels remaining 5.7–7.6-fold higher than sham controls through days 4–8.

**Conclusions:**

This exploratory study describes an optimised murine model of influenza-induced respiratory sepsis that develops biochemical and histopathological evidence of AKI. Early elevation of serum NGAL was observed in severely affected animals, supporting its potential as a candidate early biomarker. The model provides a useful platform for further mechanistic and therapeutic investigation in viral respiratory infection-associated AKI.

**Supplementary Information:**

The online version contains supplementary material available at 10.1186/s42826-026-00294-6.

## Background

Sepsis affects 49 million people annually and causes 11 million deaths worldwide [1, 2]. Acute kidney injury (AKI), characterised by abrupt kidney dysfunction, is one complication of sepsis, closely linked to high mortality [3, 4]. Viral respiratory infections, fuelled by escalating community transmission of seasonal influenza and COVID-19, drive an underexplored subset of sepsis-associated AKI.

Conventional AKI diagnosis hinges on serum creatinine, a lagging and nonspecific indicator of renal impairment [5]. Neutrophil gelatinase-associated lipocalin (NGAL) is gaining recognition as an early biomarker of tubular injury, rapidly upregulated and secreted from renal epithelium in response to insults [5–7]. NGAL rises prior to creatinine alterations, enabling proactive AKI identification [8, 9]. Notably, patients with sepsis-associated AKI tend to exhibit higher plasma and serum NGAL levels than those with AKI from non-septic causes [6]. Despite its promise, the effectiveness of using NGAL as an AKI marker remains controversial [9–11].

Building on our prior established murine model of influenza-induced respiratory sepsis [12], we further optimised this platform to delineate AKI pathogenesis. We use the term “influenza-induced respiratory sepsis” because the high-dose infection resulted in clear multi-organ dysfunction (evidenced by biochemical, histopathological, and clinical parameters) in addition to severe lung inflammation, consistent with contemporary frameworks of sepsis pathophysiology [12]. Emphasis was placed on renal NGAL expression, inflammatory cytokines, and longitudinal tracking of serum NGAL to clarify the role of NGAL as a sensitive, dynamic parameter for early AKI inception and escalation. Integrated with clinical scoring, histopathological examination, and systemic inflammatory analyses, this refined model offers a useful exploratory platform for biomarker discovery and therapeutic studies in viral respiratory sepsis and its associated AKI.

## Methods

### Animal model and viral inoculation

Male BALB/c mice (eight weeks old) were used to establish a murine model of viral respiratory sepsis. Mice were intranasally inoculated with the H1N1 influenza A/PR/8/34 strain at predetermined doses of 3.7 × 10^1^, 3.7 × 10^3^, 3.7 × 10^4^ median tissue culture infectious dose (TCID50) selected based on our prior work to elicit graded severities of respiratory sepsis (*n* = 3 per dose) [12]. Control animals (sham group, *n* = 3) received phosphate-buffered saline (PBS) via the same route. All procedures were conducted under approved institutional animal care protocols. The workflow is shown in Supplementary file 1.

### Clinical monitoring and endpoint criteria

Mice were monitored daily for 14 days following viral inoculation. Clinical severity was assessed using the Murine Sepsis Score (MSS) by the fourth author (AL) and fifth author (YZ), both blinded to treatment group. Humane endpoints (HEP) were defined as ≥ 30% body weight loss, a modification from the previously used 20% threshold. This change was specifically approved by the Committee on the Use of Live Animals in Teaching and Research (CULATR approval number: 23–507) to allow sufficient time for the development of clinically relevant sepsis phenotypes while maintaining rigorous animal welfare monitoring. Hypoglycaemia was defined as blood glucose < 70 mg/dL based on published criteria in murine critical illness and sepsis models. Physiological parameters, including body weight and blood glucose levels, were recorded daily to monitor disease progression.

### Tissue collection and histological analysis

Pathological assessment served as the gold standard for confirming organ injury. Animals were euthanised either upon reaching the HEP or at the conclusion of the 14-day observation period. Organs including the heart, lungs, spleen, liver, and kidneys were harvested immediately after euthanasia. For histological evaluation, tissues were fixed in 10% neutral-buffered formalin, as previously described [12, 13]. Tissue sections were stained with haematoxylin and eosin (H&E). For each tissue from each mouse, five representative microscopic images were captured at 20× magnification. These images were analysed semi-quantitatively, as detailed in Supplementary file 2. The scoring criteria were adapted from published studies on murine sepsis and organ injury and have been consistently used in our laboratory. Histological scoring was initially performed by the first author (YJ) and independently confirmed by an experienced pathologist (JN) blinded to treatment group. Normal histological features were referenced from educational resources such as Pathweb (National University of Singapore, https://medicine.nus.edu.sg/pathweb/).

### Assessment of organ function and inflammatory markers

Blood samples were collected for biochemical analysis of systemic organ function using the Sysmex BX-3010 chemistry analyser (Sysmex, Norderstedt, Germany). Parameters measured included serum creatinine, creatinine phosphokinase (CPK), lactate dehydrogenase (LDH), and total bilirubin. Platelet counts were determined using the Sysmex XN-1000 V haematology analyser. For molecular analysis, half of the right kidney was cut into small pieces using scissors and homogenised in TRIzol reagent using a TissueRuptor II (Qiagen). The homogenate was snap-frozen in liquid nitrogen and stored at -80 °C until processing. Total RNA was extracted according to the manufacture’s protocol. RNA concentration and purity were measured using a NanoDrop spectrophotometer, and cDNA was synthesised using the High-Capacity cDNA Reverse Transcription Kit (Thermo Fisher Scientific, Cat. No. 4374966). Kidney injury and inflammation were further evaluated by quantifying the expression of NGAL and inflammatory cytokines (IL-1β, IL-6, TNF, and IL-10) via quantitative polymerase chain reaction (qPCR) using the Roche LightCycler 480 system. The primer information was shown in Supplementary file 3. Serum NGAL levels were assessed from tail-vein blood samples collected at two-day intervals to track longitudinal changes and quantified using an enzyme-linked immunosorbent assay (ELISA) kit (Thermo Fisher Scientific, Cat# EMLCN2).

### Statistical analysis

All statistical analyses were performed using GraphPad Prism (v.10 GraphPad Software, USA). Given the small sample size (n = 3 per group), data presentation was tailored as follows: column graphs (serum biochemistry, qPCR mRNA expression, and histology scores) show median with interquartile range (IQR) and individual data points as scatter dot plots; longitudinal time-course graphs (body weight loss, glycaemia, and MSS) show median with range (minimum to maximum) with connecting lines; and correlation analyses were performed using Spearman’s rank correlation coefficient with 95% confidence intervals reported as standard output. Group comparisons were conducted using the Man-Whitney U test, Student’s t-test, or Kruskal-Wallis test as appropriate. Given the exploratory nature of the study and small sample size, no correction for multiple comparisons were applied. Exact p-values are reported in the text and figures where relevant. A p-value < 0.05 was considered statistically significant. This study is considered exploratory due to the limited sample size.

## Results

### Temporal profiles of sepsis following H1N1 infection

#### Survival rate

**Fig. 1:**
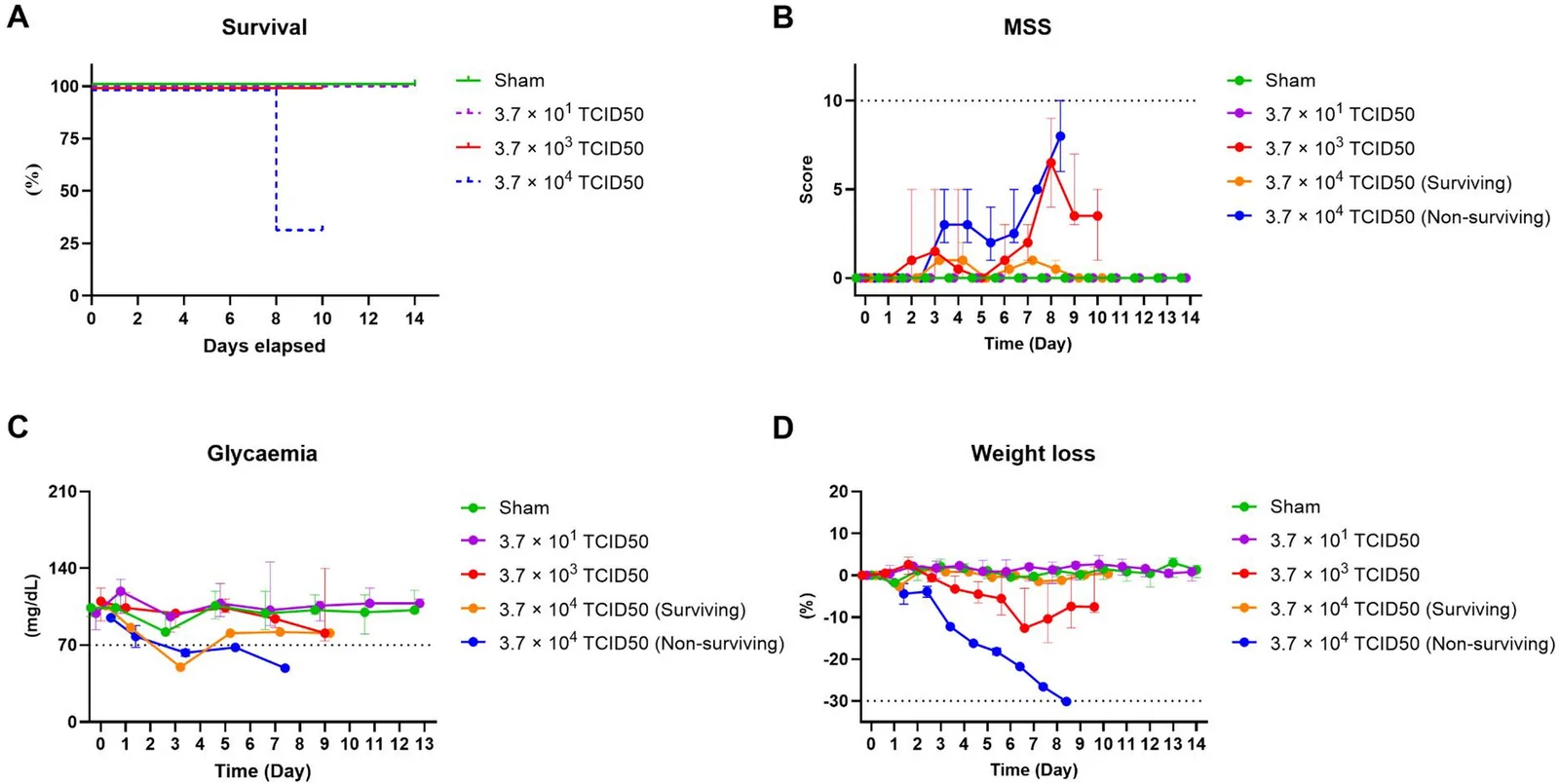
Physical conditions of 8-week-old male BALB/c mice after intranasally inoculated with influenza virus H1N1 strain A/PR/8/34. Doses of 3.7 × 10^1^, 3.7 × 10^3^ and 3.7 × 10^4^ median tissue culture infectious dose (TCID50) were used. Survival curve (**A**), Murine Sepsis Score (MSS) (**B**), glycemia (**C**), and body weight loss (**D**) were evaluated over 14 days. Sham: PBS control. *n* = 3 per group. Data are presented as median with range

Mice challenged with the highest viral dose (3.7 × 10⁴ TCID₅₀) exhibited 66.7% mortality (2 of 3 mice) by day 8 (Fig. 1A), with mice reaching the humane endpoint counted as deaths in the survival analysis. No fatalities occurred in the lower-dose groups, indicating a threshold for lethality at higher viral loads.

#### Clinical severity (MSS)

MSS increased in a dose-response manner, peaking on day 8 post-infection. An MSS of 10 was recorded in mice exposed to the highest viral dose (3.7 × 10⁴ TCID₅₀), characterised by extensive back piloerection and suppressed activity (Fig. 1B), indicative of severe systemic distress.

#### Glycaemia

Hypoglycaemia (blood glucose < 70 mg/dL) was evident from day 3, particularly in the higher dose groups, indicating metabolic dysregulation (Fig. 1C).

#### Body weight loss

Progressive weight loss was observed across all infected groups. In the 3.7 × 10⁴ TCID₅₀ group, two mice reached the humane endpoint threshold of 30% loss (Fig. 1D).

**Fig. 2:**
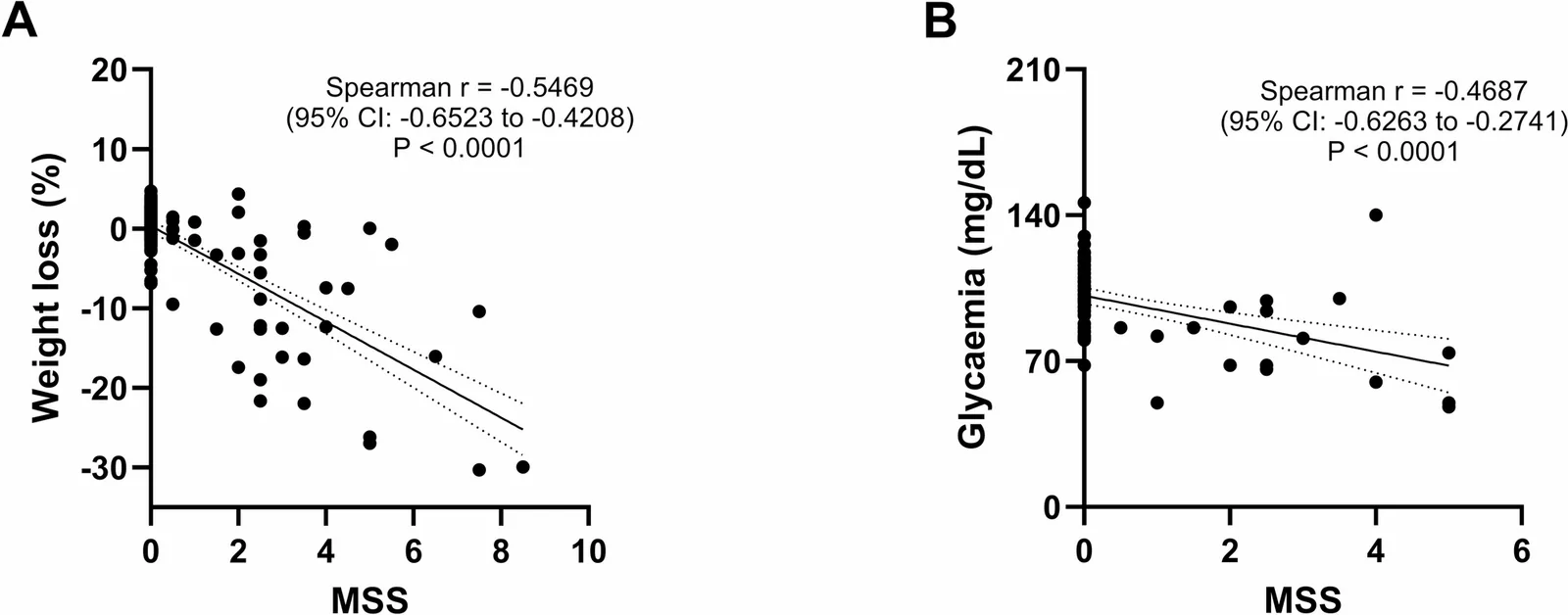
Correlation between MSS, body weight loss, and glycaemia. Correlation analyses were performed using Spearman’s rank correlation coefficient. 95% CI: 95% confidence interval

Correlation analysis revealed significant associations between MSS and body weight loss (Spearman *r* = -0.5469 [95% CI -0.6523 to -0.4208]), as well as MSS and glycaemia (*r* = -0.4687 [95% CI: -0.6263 to -0.2741]) (Fig. 2), supporting the utility of these parameters in tracking viral respiratory sepsis severity.

### Biochemical assessment of organ dysfunction

#### Kidney function

**Fig. 3:**
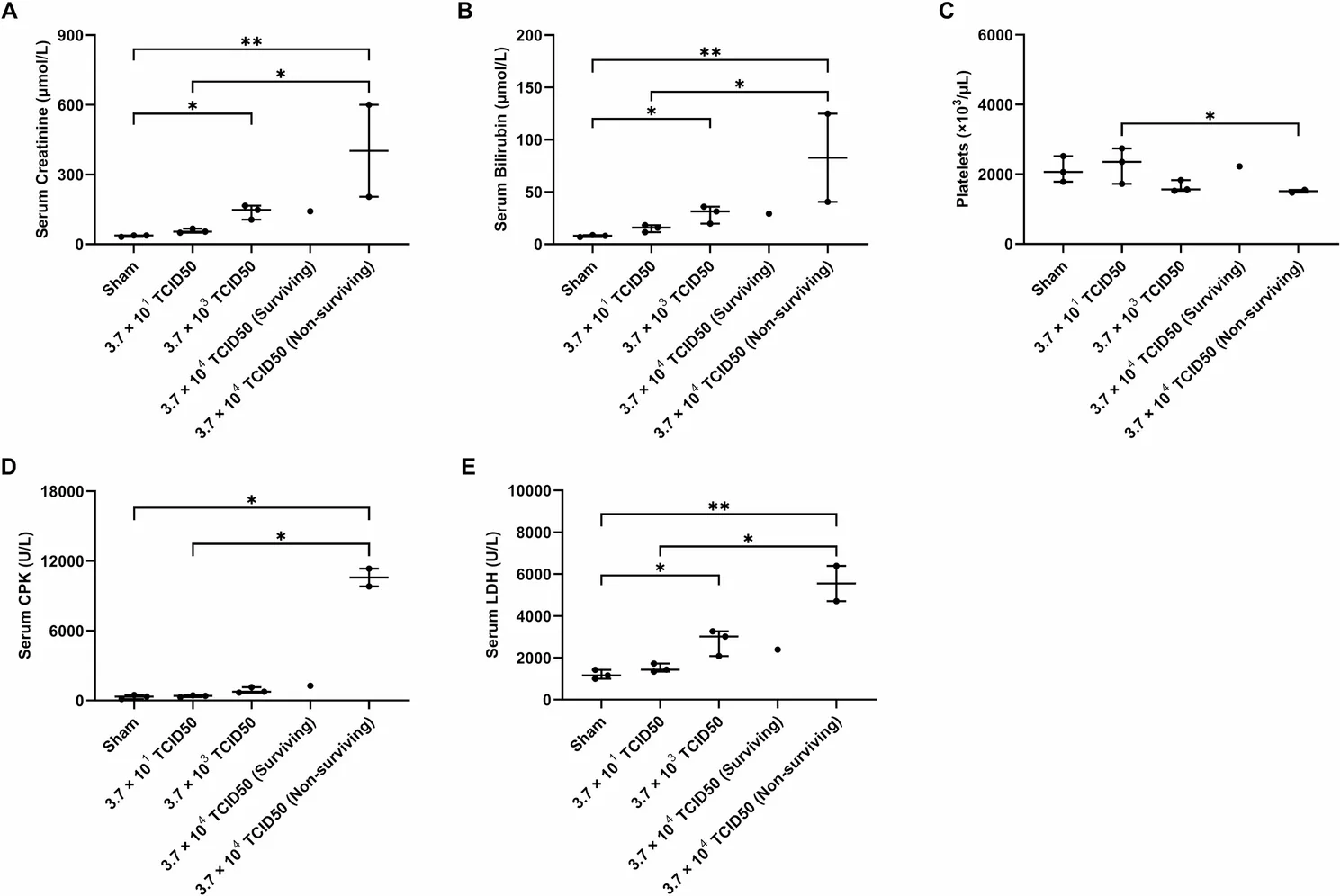
Influenza virus-induced organ dysfunction assessed by serum biomarkers. Mice were intranasally administrated influenza A virus H1N1 strain A/PR/8/34 at doses of 3.7 × 10^1^, 3.7 × 10^3^, or 3.7 × 10^4^ median tissue culture infectious dose (TCID50), or sham-treated with PBS (control). Shown are serum levels of (**A**) creatinine (renal function), (**B**) bilirubin (liver function), (**C**) platelet counts (coagulopathy), (**D**) creatinine phosphokinase (CPK; cardiac muscle injury), and (**E**) lactate dehydrogenase (LDH; systemic tissue injury/inflammation). Data are presented as medians with interquartile range (IQR); *n* = 3 per group. Statistical analysis: Kruskal-Wallis test with uncorrected Dunn’s multiple comparisons (asterisks indicate significant comparisons only: * *P* < 0.05; ** *P* < 0.01)

Serum creatinine levels remained low and stable in sham mice (median: 38 [IQR: 32–38] µmol/L). In infected mice, levels increased in a dose-dependent manner, with values of 54 [IQR: 50–67], 148 [IQR: 106–166], and 204 [IQR: 142–600] µmol/L across ascending H1N1 doses (Fig. 3A; Supplementary file 4). Based on the proposed murine-SOFA creatinine criteria (Supplementary file 5), individual mice in the 3.7 × 10^3^ and 3.7 × 10^4^ TCID50 groups scored from 1 to 4 for renal dysfunction, whereas all sham mice scored 0 (Supplementary file 6).

#### Liver function

Serum bilirubin levels also rose progressively with viral doses. Sham mice exhibited a median of 8.15 [IQR: 6.86–8.67] µmol/L. Infected mice showed elevations to 15.93 [IQR: 11.62–18.18], 31.38 [IQR: 19.68–35.84] and 40.62 [IQR: 29.3–124.9] µmol/L for the 3.7 × 10¹, 3.7 × 10³, and 3.7 × 10⁴ TCID₅₀ groups, respectively (Fig. 3B; Supplementary file 4). Four mice scored 1 and one scored 2 on the murine-SOFA bilirubin criterion, indicating liver dysfunction (Supplementary file 6).

#### Coagulation

Platelet counts declined slightly only in the highest dose group (1,554 [IQR: 1,477-2,226] x10^3^/µL) compared with sham (2,066 [IQR: 1,786–2,520] x10^3^/µL) (Fig. 3C; Supplementary file 4). All mice scored 0 on the murine-SOFA platelet criterion, suggesting no significant coagulopathy (Supplementary file 6).

#### Cardiac and systemic injury

Serum CPK levels increased markedly in response to escalating viral doses (Fig. 3D; Supplementary file 4), compared with low levels in sham mice (336.0 [IQR: 130–465] U/L). In the 3.7 × 10⁴ TCID₅₀ group, hyperCPKemia (> 10,000 U/L) was observed, consistent with significant muscle injury that may include cardiac involvement. LDH levels followed a similar trend (Fig. 3E; Supplementary file 4).

Overall, biochemistry parameters of creatinine, bilirubin, platelets, CPK, and LDH reflected the onset of multiple organ dysfunction after influenza H1N1 infection.

### Histopathological findings of tissue damage

#### Lung

**Fig. 4:**
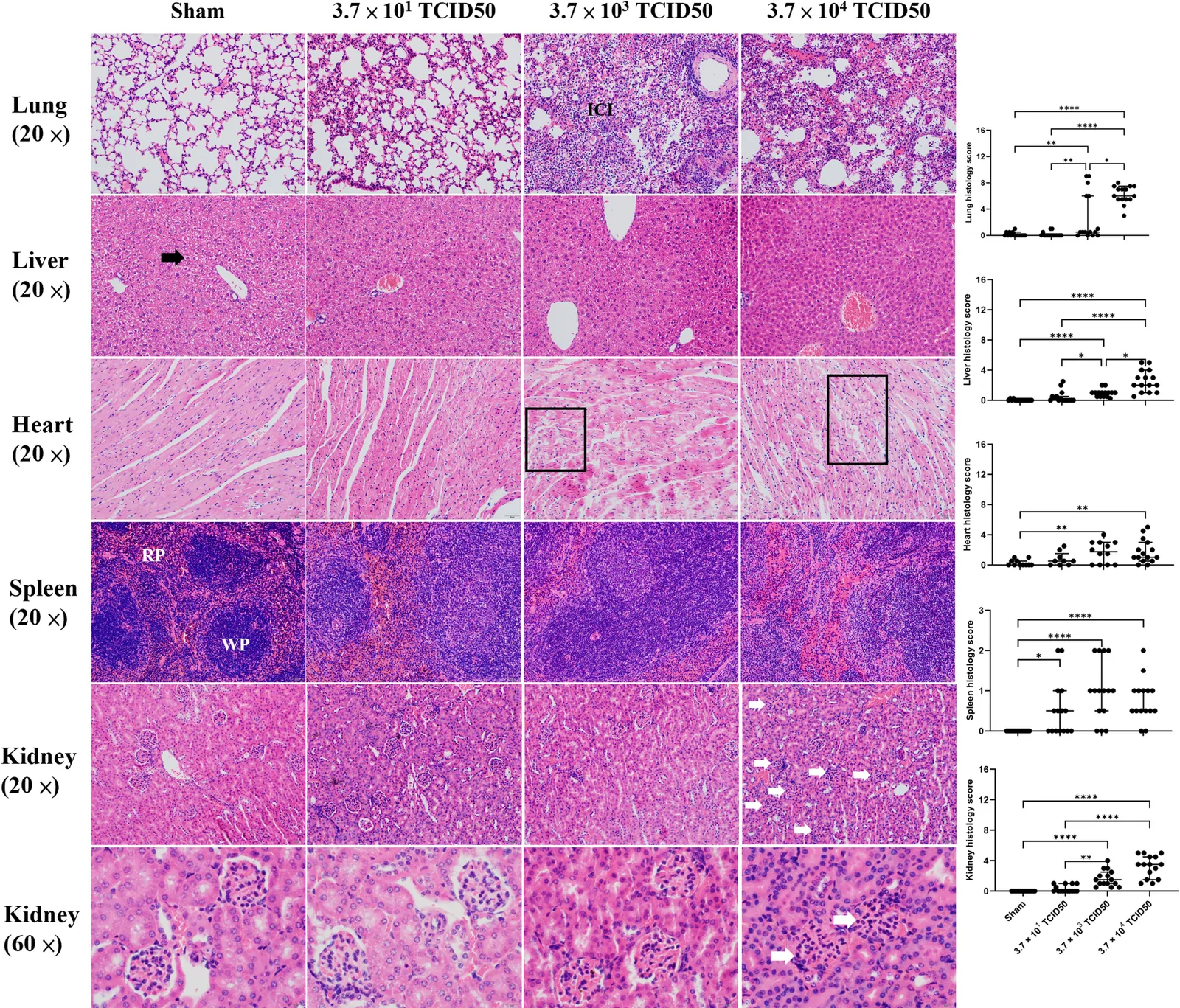
Histopathological changes in multiple organs of sham (PBS control) and influenza A/PR/8/34 (H1N1) infected mice. Representative hematoxylin and eosin (H&E)-stained sections of lung, liver, heart, spleen, and kidney are shown. Key features are indicated: ICI, inflammatory cellular infiltration; white arrow, enlarged glomeruli; black arrow, hepatocytes containing abundant glycogen; black frame, wavy myocardial fibres; RP, red pulp; WP, white pulp. All images were acquired at 20× magnification. For the kidney, additional images at 60× magnification are provided to highlight glomerular enlargement. Histology scores for each organ were compared using the Kruskal-Wallis test followed by uncorrected Dunn’s post hoc test. Data are presented as medians with interquartile range (IQR); *n* = 3 mice per group, with 5 representative images analysed per tissue per mice. Statistical significance is indicated only for comparisons reaching significance (* *P* < 0.05, ** *P* < 0.01, *** *P* < 0.001, **** *P* < 0.0001)

Lung tissues from sham mice exhibited classic thin alveolar septa (Fig. 4), indicative of normal pulmonary architecture and efficient respiratory gas exchange. In contrast, mice infected with H1N1 at doses of 3.7 × 10³ TCID₅₀ and 3.7 × 10⁴ TCID₅₀ showed extensive inflammatory cellular infiltration, reflecting severe lung inflammation and injury following H1N1 invasion.

#### Kidney

Sham mice displayed normal glomeruli with clear Bowman’s spaces. In contrast, mice receiving the highest viral dose (3.7 × 10⁴ TCID₅₀) exhibited glomeruli enlargement, tubular degeneration, narrowed tubular lumens, and increased cellularity, suggesting kidney injury and impaired renal function (Fig. 4).

#### Liver

Hepatic tissues from sham mice demonstrated abundant glycogen accumulation within hepatocytes. In mice infected with 3.7 × 10⁴ TCID₅₀, there is a notable depletion of glycogen within hepatocytes, accompanied by cytoplasmic vacuolisation, indicative of hepatocellular stress and metabolic disruption.

#### Heart

Cardiac tissues from infected mice (3.7 × 10³ TCID₅₀ and 3.7 × 10⁴ TCID₅₀) revealed wavy cardiac muscle fibres, a hallmark of myocardial infarction which may be secondary to hypoxia resulting from severe lung inflammation.

#### Spleen

In sham mice, spleen architecture was well-preserved, with clearly defined white pulps (WP) and surrounding red pulps (RP). In H1N1 infected mice, the white pulp was expanded by increased numbers of lymphocytes, and the boundary between WP and RP appeared blurred, likely reflecting an active immune response to viral infection.

### Kidney injury and inflammatory response

#### NGAL expression

**Fig. 5:**
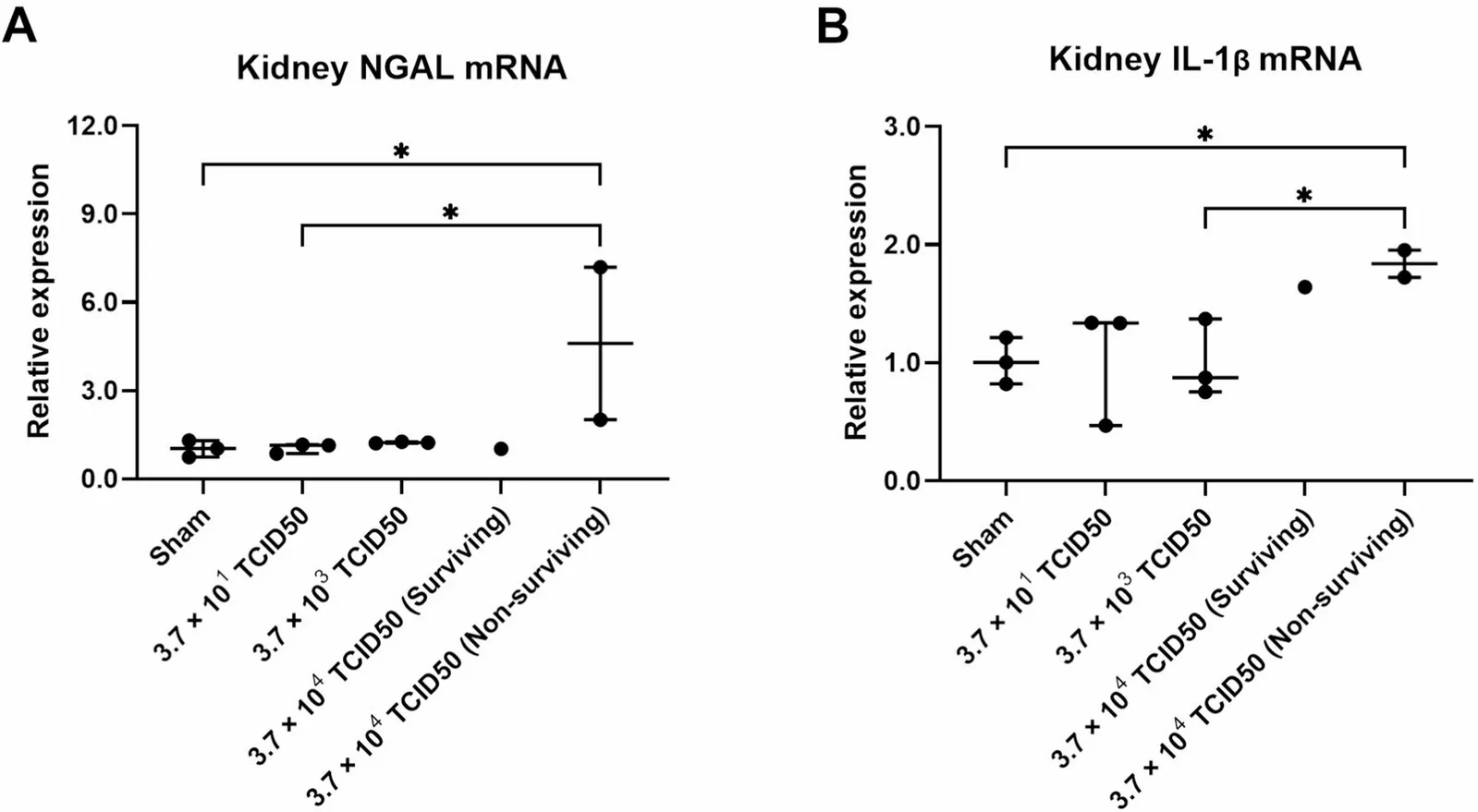
Quantitative real-time PCR (qPCR) analysis of NGAL (**A**) and IL-1β (**B**) mRNA expression in mouse kidneys following influenza A/PR/8/34 (H1N1) infection. Mice were intranasally inoculated with influenza A/PR/8/34 (H1N1) virus at doses of 3.7 × 10¹, 3.7 × 10³, and 3.7 × 10⁴ TCID₅₀. Kidney tissues were collected at the humane endpoint (HEP) or on day 14 post-infection. mRNA levels were normalised to GAPDH and relative expression was calculated using the ΔΔCt method. Data are shown as median with interquartile range (IQR; *n* = 3 per group). Statistical analysis: Kruskal-Wallis test with uncorrected Dunn’s multiple comparisons (asterisks indicate significant comparisons only: * *P* < 0.05)

Kidney NGAL mRNA levels were significantly elevated in influenza-infected mice, with 2-fold and 7-fold increases observed in two non-surviving mice at the highest dose (3.7 × 10⁴ TCID₅₀; *P* = 0.0483) (Fig. 5A). This upregulation positively correlated with serum creatinine levels, supporting virus-induced kidney injury.

#### IL-1β expression

Kidney IL-1β mRNA levels were also significantly increased in the highest dose group (Median: 1.72 [IQR: 1.64–1.95]; *P* = 0.0315) (Fig. 5B). As IL-1β is a known driver of renal NGAL production [16], its elevation at day 8 post-infection supports ongoing injury and repair processes.

#### Other cytokines

IL-6, TNF-α, and IL-10 were undetectable in kidney tissue across all groups, possibly due to tissue-specific immune responses or the timing of sample collection. The kidney, not being a primary target of influenza A virus, may exhibit minimal systemic cytokine expression in the absence of secondary inflammation.

### Temporal changes in serum NGAL levels

**Fig. 6:**
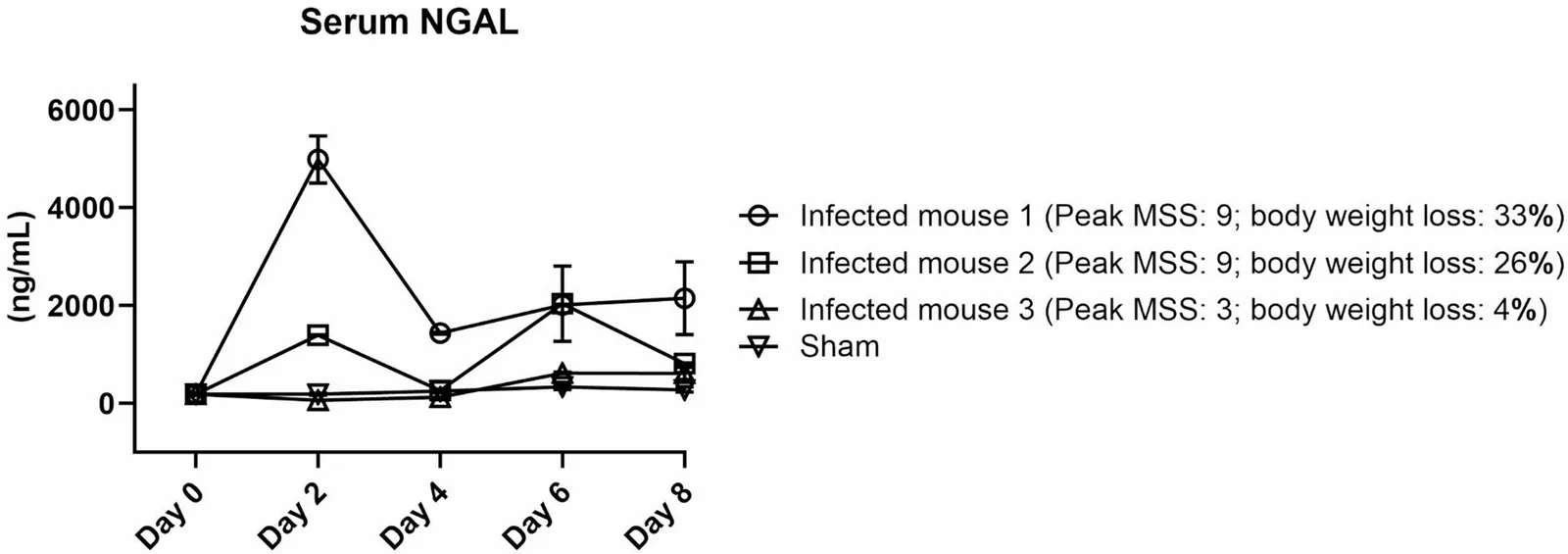
Longitudinal changes in serum NGAL levels in individual mice following influenza A/PR/8/34 (H1N1) infection. Mice (*n* = 3) were intranasally inoculated with 3.7 × 10⁴ TCID₅₀ of influenza A/PR/8/34 (H1N1) virus. Infected mice exhibited variable disease severity, with peak Mouse Sepsis Score (MSS) of 9 (mouse 1; 33% body weight loss), 9 (mouse 2; 26% body weight loss), and 3 (mouse 3; 4% body weight loss). Each line represents repeated measurements from a single individual mouse obtained via serial tail-vein sampling. Serum NGAL levels were measured in duplicate by ELISA. Data are presented as median with range

Serum NGAL levels were low at baseline (median 191 ng/mL; IQR 169–212). In the high-dose group, longitudinal monitoring via serial tail-vein sampling was performed in mice that survived beyond day 2. Each line in Fig. 6 represents repeated measurements from a single individual mouse. A sharp rise to 4,990 ng/mL on day 2 was observed in the most severely affected mouse (peak MSS: 9; 33% body weight loss). Another mouse showed a moderate increase to 1,399 ng/mL (peak MSS: 9; 26% body weight loss), while the least affected mouse (peak MSS: 3; 4% body weight loss) showed no notable rise (Fig. 6). In the two mice with elevated NGAL on day 2, levels subsequently declined but remained substantially elevated (5.7–7.6-fold higher) compared with sham controls through days 4–8 (Fig. 6). These preliminary individual trajectories illustrate the potential of serum NGAL as an early and dynamic biomarker of AKI in influenza-induced respiratory sepsis.

## Discussion

We describe an exploratory optimisation of a murine model of influenza-induced respiratory sepsis that produces biochemical, histopathological, and molecular evidence of kidney injury. This model also shows early elevation of serum NGAL in severely affected animals.

The highest viral dose (3.7 × 10⁴ TCID₅₀) induced 66.7% mortality (2 of 3 mice) by day 8, which lies within the broad clinical range reported for severe respiratory sepsis (30% − 70%) [14, 15]. Dose-dependent increases in clinical severity score, body weight loss, hypoglycaemia, and multi-organ dysfunction were observed.

Extending the HEP criterion for body weight loss from 20% to 30% allowed fuller manifestation of organ dysfunction and sepsis phenotypes before euthanasia. This refinement also enabled detection of dose-dependent effects, which were not observed in our previous study using the 20% threshold [12].

Kidney injury was evidenced by elevated serum creatinine, glomerular enlargement, tubular degeneration, narrowed tubular lumens, and increased cellularity in high-dose infected mice. These histopathological changes, particularly the tubular degeneration, were accompanied by a 7-fold upregulation of renal NGAL mRNA expression, consistent with NGAL’s established role as a sensitive marker of tubular epithelial injury. IL-1β mRNA was also significantly increased. IL-1β is a known driver of renal NGAL production, and its elevation detected at day 8 indicates ongoing local inflammation and tissue repair processes [16]. Other cytokines (IL-6, TNF, and IL-10) were undetectable in kidney tissue at day 8 post-infection. This absence likely reflects the temporal dynamics that IL-6 and TNF typically peak within hours of stimulation and decline rapidly due to short half-lives and tight regulation [17–20]. IL-1β can persist longer due to continuous processing and release via the inflammasome in response to ongoing cellular stress or damage, thus contributing to more sustained tissue responses in the kidney microenvironment [21]. Additionally, influenza A virus primarily targets the respiratory tract, so renal cytokine expression may remain minimal without direct viral invasion. AKI in this model therefore probably arises indirectly via hypoxaemia from severe lung inflammation, cardiac compromise, oxidative stress, or systemic metabolic dysregulation [22]. The observed multi-organ pathology including lung infiltration, myocardial wavy fibre, hepatic glycogen depletion, and splenic lymphoid hyperplasia, further supports interconnected mechanisms that amplify kidney injury.

Compared with classical caecal ligation and puncture (CLP) or lipopolysaccharide (LPS) models that predominately simulate peritoneal or endotoxin-driven bacterial sepsis [23], this influenza model offers distinct advantages. It employs a natural respiratory route of viral infection and produces a more gradual disease progression, thereby better mimicking community-acquired viral respiratory sepsis commonly encountered in emergency departments [24]. We additionally applied a novel murine-SOFA scoring system, originally developed by our group as an exploratory adaptation of the human SOFA criteria. Unlike the primarily qualitative and subjective murine sepsis scoring tool (MSS), this proportional adaptation incorporates objective biochemical parameters (creatinine, bilirubin, platelets, and CPK). However, because it has not been independently validated, the scores should be interpreted cautiously. Further validation and refinement of this scoring tool will be pursued in future studies.

One key limitation of this study is the small blood volume obtainable via serial tail-vein sampling (~ 10 µL, yielding ~ 4 µL serum). This volume was sufficient for sensitive assays such as NGAL ELISA but insufficient for serum creatinine measurement, which typically requires > 100 µL serum and thus necessitates terminal bleeding. Consequently, creatinine was measured only at the experimental endpoint. This constraint precluded longitudinal monitoring of serum creatinine in individual animals, preventing direct within-subject comparisons of creatinine and NGAL dynamics. Blood urea nitrogen (BUN) was not assessed. Nevertheless, the early and marked elevation of serum NGAL (as early as day 2 post-infection) in a representative severely affected animal supports its potential as a sensitive and dynamic biomarker of AKI in influenza-induced sepsis. Future work should explore methods that enable simultaneous serial measurement of NGAL and creatinine. Such combined monitoring could provide a more nuanced picture of AKI progression [25–28].

Furthermore, while renal NGAL mRNA was upregulated, elevated serum NGAL may partly originate from extra-renal sources such as inflamed lung tissue or systemic neutrophils. Because viral load in the kidney was not quantified, we cannot fully exclude minor direct viral effects; however, the histopathological pattern and absence of widespread renal inflammation suggest that renal injury is most likely indirect.

This remains an exploratory dose-ranging study with small group sizes (*n* = 3 per group). We acknowledge that this sample size provides limited statistical power and that high biological variability (particularly in the high-dose group) restricts the generalisability and reproducibility of some findings. Future validation studies could benefit from larger group sizes (e.g., *n* = 5–8 per group for ~ 90% power) and formal a priori power calculations for each primary endpoint.

Additional limitations include the absence of more detailed cytokine profiling, mechanistic studies on NGAL regulation, and viral load quantification in the kidney. Future work should include validation of serum NGAL in clinical cohorts of viral respiratory infections and evaluation of therapeutic interventions using this model.

## Conclusions

This exploratory study presents an optimised murine model of influenza-induced respiratory sepsis that develops multi-organ dysfunction including AKI. Early increases in serum NGAL were observed in severely affected animals. While larger confirmatory studies are needed, the model offers a useful platform for investigating viral respiratory infection-associated AKI and candidate biomarkers such as NGAL.

## Supplementary Information

Below is the link to the electronic supplementary material.

Supplementary Material 1

## Data Availability

All datasets, on which the conclusions of the manuscript rely on, are presented in the paper.

## Abbreviations

AKI: Acute kidney injury
BALB/c: Bagg albino laboratory-bred/c
CI: Confidence interval
CPK: Creatine phosphokinase
H&E: Haematoxylin and eosin
HEP: Humane endpoint
IL-1β: Interleukin-1 beta
IL-6: Interleukin-6
IL-10: Interleukin-10
LDH: Lactate dehydrogenase
MSS: Murine sepsis score
NGAL: Neutrophil gelatinase-associated lipocalin
PBS: Phosphate-buffered saline
qPCR: quantitative polymerase chain reaction
SD: Standard deviation
SOFA: Sequential organ failure assessment
TCID50: Median tissue culture infectious dose
TNF: Tumour necrosis factor
