# Supplementary material for: Optimised murine model of influenza-induced respiratory sepsis for studying acute kidney injury
Source: Lab Anim Res. 2026 Aug 26;42:31. doi: 10.1186/s42826-026-00294-6 (PMC13508304; doi:10.1186/s42826-026-00294-6)
Supplement: Supplementary file 1 [file 42826_2026_294_MOESM1_ESM.docx]

**Supplementary file 1** Workflow for constructing an influenza-induced respiratory sepsis model and assessing AKI via dynamic profiling of serum NGAL.

**Supplementary file 2** Semi-quantitative scoring systems for histological analysis.

| **Lung** | None  = 0 | | Minimal  = 1 | | Mild  = 2 | | Significant  = 3 | | Severe  = 4 |
| --- | --- | --- | --- | --- | --- | --- | --- | --- | --- |
| 1. Edema in the alveolar spaces |  | |  | |  | |  | |  |
| 2. Thickness of the alveolar wall |  | |  | |  | |  | |  |
| 3. Infiltration of inflammatory cells |  | |  | |  | |  | |  |
| 4. Haemorrhage |  | |  | |  | |  | |  |
| Sum score |  | | | | | | | | |
|  |  |  |  |  |  |  |  |  |  |
| **Kidney** | None  = 0 | | Minimal  = 1 | | Mild  = 2 | | Significant  = 3 | | Severe  = 4 |
| 1. Tubular degeneration |  | |  | |  | |  | |  |
| 2. Glomerular enlargement |  | |  | |  | |  | |  |
| 3. Infiltration of inflammatory cells |  | |  | |  | |  | |  |
| 4. Haemorrhage |  | |  | |  | |  | |  |
| Sum score |  | | | | | | | | |
|  |  | | | | | | | | |
| **Heart** | None  = 0 | | Mild  = 1 | | Moderate  = 2 | | Strong  = 3 | | Intense  = 4 |
| 1. Wavy cardiac muscle fiber |  | |  | |  | |  | |  |
| 2. Necrosis of cardiomyocytes |  | |  | |  | |  | |  |
| 3. Infiltration of inflammatory cells |  | |  | |  | |  | |  |
| 4. Haemorrhage |  | |  | |  | |  | |  |
| Sum score |  | | | | | | | | |
|  |  |  |  |  |  |  |  |  |  |
| **Liver** | None  = 0 | | | Mild  = 1 | | Moderate  = 2 | | Severe  = 3 | |
| 1. Glycogen depletion |  | | |  | |  | |  | |
| 2. Cytoplasmic vacuolisation |  | | |  | |  | |  | |
| 3. Infiltration of inflammatory cells |  | | |  | |  | |  | |
| 4. Haemorrhage |  | | |  | |  | |  | |
| Sum score |  | | | | | | | | |

| The degree of **splenic** injury | **Score** |
| --- | --- |
| Normal morphology | 0 |
| Mild disorder of white pulp, characterised by local hyperplasia | 1 |
| Moderate disorder of white pulp, blurring of the boundary between white pulp and red pulp, and the white pulp structures like follicles and marginal zones may vanish | 2 |
| High disorder of white pulp, almost no significant difference between white pulp and red pulp | 3 |

**Supplementary file 3** qPCR primers

|  | **Forward (5'->3')** | **Reverse (5'->3')** | **Product length** | **NCBI Ref. Seq.** |
| --- | --- | --- | --- | --- |
| **NGAL** | ATGTCACCTCCATCCTGGTCAG | GCCACTTGCACATTGTAGCTCTG | 145 | NM_008491.1 |
| **GADPH** | CATCACTGCCACCCAGAAGACTG | ATGCCAGTGAGCTTCCCGTTCAG | 153 | NM_008084.4 |
| **IL-1β** | TGGACCTTCCAGGATGAGGACA | GTTCATCTCGGAGCCTGTAGTG | 148 | NM_008361.4 |
| **IL-6** | TACCACTTCACAAGTCGGAGGC | CTGCAAGTGCATCATCGTTGTTC | 116 | NM_031168.2 |
| **IL-10** | CGGGAAGACAATAACTGCACCC | CGGTTAGCAGTATGTTGTCCAGC | 130 | NM_010548.2 |
| **TNF** | GGTGCCTATGTCTCAGCCTCTT | GCCATAGAACTGATGAGAGGGAG | 139 | NM_013693.3 |

**Supplementary file 4** Summary of individual mouse data across experimental groups and control group in H1N1 viral sepsis study.

| **Group** | **Mouse no.** | **Platelet counts, x10^3^/µL** | **Serum creatinine, μmol/L** | **Serum bilirubin, μmol/L** | **Serum CPK, U/L** | **Serum LDH, U/L** |
| --- | --- | --- | --- | --- | --- | --- |
| **Sham (PBS control)** | **A1** | 2520 | 38 | 8.67 | 465 | 1432 |
|  | **A2** | 2066 | 32 | 6.86 | 336 | 1166 |
|  | **A3** | 1786 | 38 | 8.05 | 130 | 1005 |
| **3.7 × 10^1^ TCID50** | **B1** | 2357 | 54 | 15.93 | 438 | 1342 |
|  | **B2** | 1726 | 67 | 18.18 | 297 | 1436 |
|  | **B3** | 2742 | 50 | 11.62 | 401 | 1732 |
| **3.7 × 10^3^ TCID50** | **C1** | 1526 | 166 | 35.84 | 1148 | 3274 |
|  | **C2** | 1834 | 148 | 31.38 | 764 | 3018 |
|  | **C3** | 1568 | 106 | 19.68 | 680 | 2086 |
| **3.7 × 10^4^ TCID50** | **D1** | 1554 | 600 | 124.92 | 9822 | 6396 |
|  | **D2** | 1477 | 204 | 40.62 | 11352 | 4716 |
|  | **D3** | 2226 | 142 | 29.30 | 1272 | 2398 |

**Supplementary file 5** Proposed murine-SOFA scoring criteria of platelets, bilirubin, creatinine, and CPK in assessing murine sepsis.

| **System Parameters** | **0** | **1** | **2** | **3** | **4** |
| --- | --- | --- | --- | --- | --- |
| **Renal** Creatinine, μmol/L | Normal (0 – 120) | 121 – 187 | 188 – 329 | 330 – 440 | > 440 |
| **Liver** Bilirubin, μmol/L | Normal (0 – 28) | 29 – 46 | 47 – 144 | 145 – 290 | > 290 |
| **Coagulation** Platelets, x10^3^/µL | Normal (≥ 1000) | < 1000 | < 667 | < 333 | < 133 |
| **Cardiac** CPK, U/L | Normal (0 - 648) | 649 - 1296 | 1297 - 1944 | 1945 - 2592 | >2592 |

Note: The ranges for each criterion were assigned by proportion corresponding to human SOFA.

**Supplementary file 6** Murine-SOFA score assessment in sham and influenza-infected mice. Organ dysfunction was evaluated using a modified murine-SOFA scoring system based on proportional thresholds derived from the human SOFA criteria. Score of serum creatinine (A), serum bilirubin (B), platelet counts (C), and serum creatinine phosphokinase (CPK) (D).
